# The Western Tripod of Medicine

**Published:** 1868-08-01

**Authors:** 


### EDITORIAL.

### *"The Western Tripod of Medicine."*

UNDER this caption the *Leavenworth Medical Herald* bewails the unhappy results of the low fees of the Medical Colleges in Cincinnati and Chicago, as contrasted with the high fees, nominally, charged in St. Louis. The amiable writer is evidently sincere in his opinions, but he must pardon us for saying, "he has got hold of his pitcher by the wrong handle."

All his article is based upon two assumptions, neither of which (he must excuse us for saying), is supported by the facts.

The first assumption is contained in the following paragraphs:

> "We have hitherto confined our remarks in great measure to the low standard of the doctorate in our country, and the utter absence of that uniform grade of acquirement, which should be universally and peremptorily demanded as the just equivalent of a most honorable distinction. Though the great mass of the medical profession, appreciating the debased level to which the shallow flooding by the colleges must reduce their noble calling, have been actively agitating the question of a wholesome reform, yet the professional ambition and the collegiate rivalry have resulted in the unnecessary multiplication of schools, and in some instances, in an unworthy and almost commercial strife for patronage.
>
> As though it were not bad enough, however, to open the portals of a most intricate and profound science to whomever may present himself with the nummulary password, regardless of any intrinsic adaptation to, or acquired fitness for, the exalted position to which he aspires, some of our western schools, by reducing the feeble barrier erected by a reasonable monetary requirement, have almost obliterated the last obstacle to a universal flooding of the ranks medical; and have made the creation of a modern doctor of medicine but the question of some fifteen months of time, with the expenditure of about one hundred dollars in money."

To which, the present writer, as one who has been connected with Medical Colleges nearly the entire time for the last quarter of a century, respectfully replies, that the mere fact of the presence or absence of fees has no logical or practical relation to the character of the lectures in either of the colleges alluded to, or in any other college he has ever known. The matter of high or low fees has no logical or practical relation to the standard of qualifications required for the doctorate here or elsewhere.

It is a novel idea that increasing the opportunities of young men to acquire professional or any other knowledge, is in the nature of things an encouragement for ignorance. If so, our magnificent system of primary schools, academies and colleges in this country had better be abandoned at once. As a matter of history, low as may be the *status* of the profession at the present time, it has wonderfully improved since the introduction of medical colleges in the North-west. The colleges may not have brought up the profession to the high point of attainment deemed practicable by some enthusiastic optimists, but they have accomplished a great work already, and are steadily advancing its best interests.

We will not speak for Rush College for obvious reasons, but let its 1,200 alumni speak for it if needed—as it is not. But we will vouch for Cincinnati, with whose teachers Chicago has none but the most amicable competition, that it affords as good lectures, and turns out as well qualified graduates as any school in the Union, with double or triple its fees.

The second assumption is (by necessary inference), that the low fees charged by the colleges in this city and Cincinnati are the result of their mutual competition, and it is intimated that both parties are ashamed of it, and each disclaims the responsibility. Although not one of the "distinguished" gentlemen to whom the *Herald* refers, we can satisfy its anxiety for information by informing it that neither Chicago nor Cincinnati are responsible. The *Marplot* in the fee business was the (now moribund) Medical Department of the University of Michigan at Ann Arbor, which swept out of existence, at once, all fees, except a trivial one for matriculation. No perfection of lectures or clinical advantages could compete with this *enfant terrible.* The names of world-wide celebrities would have counted for nothing with the swarms that went greedily down to that home of the prophets. They "went for their diplomas and got them."

At that time the "Phantom in Black" (*Lancet*) occupied a chair in the medical college of this city, and he proposed to make that college a *free* school also. Fees every where went tumbling, or if this did not nominally occur, lavish credits were given on the ostensible fees, which amounted to the same thing, or worse. "We speak what we do know."

From the date of its re-organization, in 1859, Rush College has been anxious to restore fees to their former scale. But he knows little of business who believes that any medical college will cut its own throat to accommodate the notions of men who have no practical acquaintance with carrying on successfully an institution of the kind.

As a mere matter of business protection, all the fee-charging schools should have combined to refuse to receive the tickets, or acknowledge the diplomas of the Ann Arbor concern. This was the prime error. Its effect was continued in the absurd refusal of the so-called Teacher's convention at Cincinnati, endorsed (to get rid of it) by the American Association. That convention, perpetrating a mass of balderdash and Utopian vagaries, omitted the only practical thing they could have usefully meddled with. This, again, was the work of the "Phantom," aided by his ineffable shadow and toady in the interest of Ann Arbor.

We shall probably refer to this subject again, although our own private impression is that the best way to build up a medical college, and "elevate the profession" thereby, is to stop chattering about it and go to work. We are instigated to this "by the honorable memories clustering around our institution."

One thing more. Our earnest friend fears that the time is approaching when he shall be obliged to "advocate a trans-Atlantic diploma, as the only true evidence of medical qualification." This, with our experience, is rich. Why, Brother *Herald*, to-day a diploma from any one of the schools you inveigh against, is worth more (little as it may be), as such *evidence*, than a dozen old world parchments piled one upon another. The graduate of almost any respectable American college will secure successful practice all around him, whilst the trans-Atlantic is *un-learning* that which is but a clog and burden to him.

